# Temporomandibular joint changes in oral submucous fibrosis- A magnetic resonance imaging study

**DOI:** 10.4317/jced.54643

**Published:** 2018-07-01

**Authors:** Chinnasamy Nanthini, Sankarapandian Sathasivasubramanian, Murali Arunan

**Affiliations:** 1 Senior Lecturer- Department of Oral Medicine and Radiology, Adhiparasakthi Dental College and Hospital, Melmaruvathur, India; 2 Professor and Head- Department of Oral Medicine and Radiology, Sri Ramachandra Dental College and Hospital, Chennai, India; 3 Associate Professor- Department of Radiology and Imaging Sciences, Sri Ramachandra Medical College and Research Institute, Chennai, India

## Abstract

**Background:**

The aim of this study was to assess the Temporomandibular joint changes as a consequence of varying degrees of restricted mouth opening in Oral Submucous Fibrosis patients.

**Material and Methods:**

The study Population was divided into 2 groups namely Group C- 40 TMJ’s of 20 age and gender matched healthy controls and Group P- 40 TMJ’s of 20 OSMF patients who were further subdivided into Group II,III,IV based on their restriction in mouth opening. MRI of bilateral TMJ was obtained in closed mouth position. Disc thickness, disc length, joint space and condylar changes were assessed. The collected data was subjected to statistical analysis.

**Results:**

Disc thickness, disc length and joint space was significantly reduced in Group III and Group IV OSMF patients when compared to controls (*P*<0.05). Condylar flattening was seen in Group III (56.3%) and Group IV (50%) OSMF patients. One joint (2.5%) in Group IV had condylar flattening with erosion whereas no joints in Group II OSMF and controls had condylar flattening and erosion (*P*<0.05).

**Conclusions:**

Thus the present study has revealed statistically highly significant changes in the components of Temporomandibular joint in OSMF patients with varying degrees of restriction in mouth opening when compared to controls. Also the severity of the changes increases with increase in severity of the disease, which was found to be statistically highly significant.

** Key words:**Oral submucous fibrosis, temporomandibular joint, magnetic resonance imaging.

## Introduction

Human beings have the capacity to produce a variety of movements that require the structures of the human body to both generate and respond to forces that produce and control movement at the body’s joints. The relative mobility of the joints is determined by their architecture and tissue concentration. The bony ends of the immovable or slightly movable joints are connected by Fibrous or cartilaginous membranes (syndesmoses or synchondroses). The bony components of movable joints are enclosed completely by a synovial membrane lined joint cavity (synovial or diarthrodial joints). The musculoskeletal system in general is vulnerable to disuse and over use (1). The physical stimulus of motion is crucial for maintenance of structural and functional integrity of joints. Immobilization or restricted movement is one such cause which affects the joint secondarily and produces changes. Extensive animal studies in the literature involving immobilization or restricted movement have revealed significant changes in the synovium, muscles, bone and periarticular connective tissue (2). The homeostasis of the joint is invariably affected by lack of physical stimulus. The movement of the joint in turn facilitates movement of the synovial fluid and trans synovial nutrient flow to ligaments and cartilages. Immobilization also leads to impaired lubrication of the joint which is attributed to reduced water and glycosaminoglycans contents (3,4).

The temporomandibular joint is the articulation between the mandible and the cranium. The hyaline cartilage lines the articulating surfaces of most of the joints, however the articulating surfaces of the TMJ are lined by avascular, dense, fibrous connective tissue (5). This histological distinction has been in the notion over years that the temporomandibular Joint is a non weight bearing joint. However over the last few decades considerable amount of evidences in literature have indicated that TMJ is in fact a load bearing joint (6-8). There has been very sparse data in the literature regarding the restricted movements of jaw and its effects on TMJ, most of them being animal studies (9-11). Temporomandibular joint being a synovial joint and functions according to same natural rules as several other joints in the body. Hence the changes due to mandibular hypomobility are expected in TMJ.

Oral Submucous Fibrosis (OSMF) is regarded as a potentially malignant disorder of the oral cavity (12). Trismus is one of the classical manifestation of OSMF due to accumulation of inelastic fibrous tissue in the juxta epithelial region of oral mucosa with associated muscle degeneration (13). Progressive restriction in movement of the joint secondary to OSMF and a deficient functional stimulation of TMJ leads to temporomandibular joint disuse. Keeping in mind the various degrees of immobilization of the temporomandibular joint and lack of functional stimulation to the TMJ, this study was tailored to assess the temporomandibular Joint changes in Oral submucous Fibrosis patients by Magnetic Resonance Imaging. The objectives of the study were to assess and compare the MRI findings of temporomandibular joint in OSMF patients with restricted mouth opening with that of healthy controls and to find out if there any correlation exists between the restriction in mouth opening and changes in various components of TMJ.

## Material and Methods

The present study was conducted in the Department of Oral Medicine and Radiology, Faculty of Dental Sciences, Sri Ramachandra University, Chennai, India. The study was approved by the Ethics Committee for student’s proposals, Sri Ramachandra University. Patients willing to participate in the study were explained about the study in detail and a written consent was obtained.

The study Population was divided into 2 groups namely Group C- control population and Group P- patient population. Group C consisted of 40 TMJ’s of 20 age and gender matched healthy controls without any history of tobacco or pan chewing and whose mouth opening was greater than 35mm. Group P consisted of 40 TMJ’s of 20 newly diagnosed and histologically proven OSMF patients.

Pregnant women, Individuals with parafunctional habits, any other pathological causes which leads to difficulty in mouth opening, patients with Temporomandibular joint disorders, debilitating systemic diseases, generalized degenerative joint disease, those with metallic implants, cardiac pacemakers, Prosthetic Heart Valves, Aortic stents, cochlear implants formed the exclusion criteria for both Group C and Group P.

A thorough clinical examination of the study population was carried out which included examination of oral cavity for findings such as blanching, hyper or hypo pigmentation, presence of ulcerations, tongue movements, shrunken or atrophic uvula. The palpatory findings included evaluation for fibrous vertical bands in the buccal mucosa and circum oral bands. Interincisal opening was measured from the incisal edge of upper incisors to the incisal edge of lower incisors at maximal mouth opening with the help of divider and scale. Based on the interincisal opening ,the Group P was further divided into subgroups: Group I (No limitation in mouth opening), Group II (interincisal distance of 26mm to 35 mm), Group III (Interincisal distance 15 mm to 25 mm), Group IV (Interincisal distance <15mm) according to the clinical criteria by Khanna et al 1995 (14). Group II, Group III and Group IV OSMF patients who were having limitation in mouth opening formed the core of the study group where as patients in Group I (>35mm) who did not have limitation in mouth opening was used as cut off point. All the subjects in Group C and Group P were subjected to a detailed clinical examination of TMJ using a structured questionnaire, following which each of them were subjected to MRI of temporomandibular Joint.

MRI was performed on a 1.5 Tesla Magnetom Avanto MR unit (Siemens, Erlangen, Germany) by using a 12-channel head coil (Magnetom Avanto). T1[repetition time msec/echo time msec -367/9], T2[repetition time msec/echo time msec - 2980 / 115, slice thickness - 3 mm] and Proton Density (PD) weighted images were obtained in axial, coronal and oblique sagittal sections. A section thickness of 3 mm and an intersection gap of 0.3 mm, a matrix of 512 × (305–512), and a field of view of 140–160 mm were used for all the sequences. Oblique sagittal Proton Density weighted images were utilized to evaluate the disc, joint space. Oblique sagittal T1 weighted images for evaluating morphology of the condyles. Imaging was done in closed mouth with the mandible in relaxed position without clenching. The following components in the TMJ were then evaluated in the work station.

**Figure 1:**
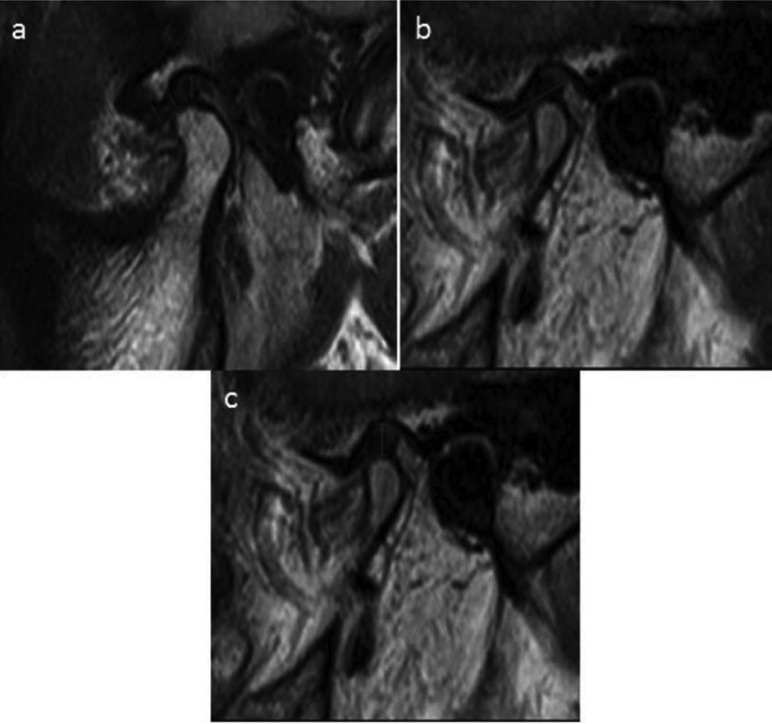
a) Measurement of thickness of articular disc. Red lines indicate anterior band, intermediate zone and posterior band. b) Measurement of disc length. c) Measurement of joint space.

**Figure 2:**
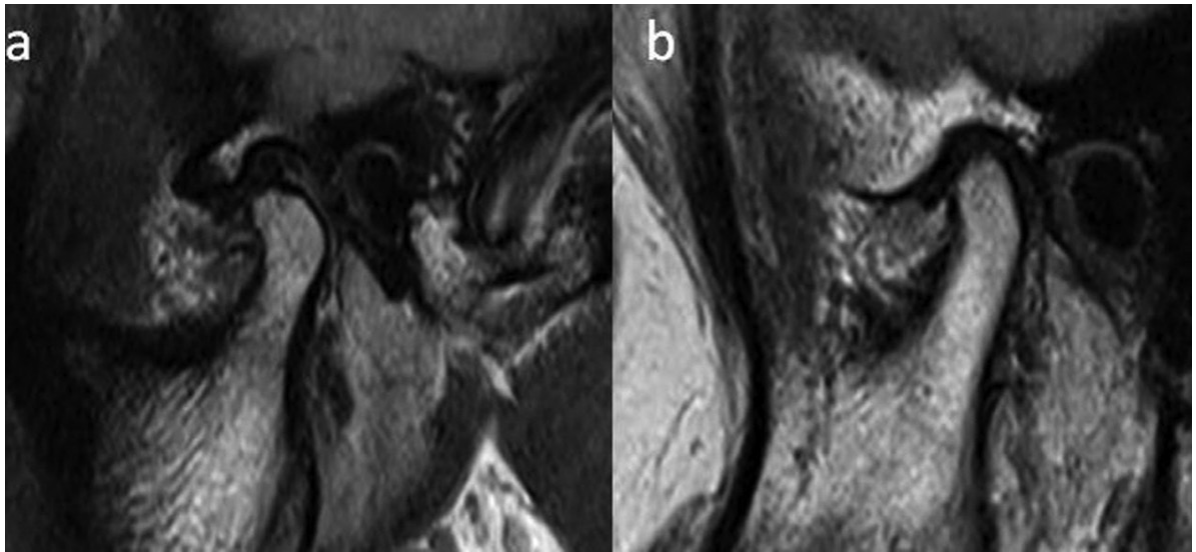
a): MRI of control showing ideal biconcave articular disc and normal joint space. b): MRI of OSMF patient showing thinning of disc and narrowing of joint space.

-Thickness of the Disc:

The thickness of the disc was measured at the level of anterior band, intermediate zone and posterior band on the sections where the discs were best seen (Fig. 1a, 2).

-Disc Length:

Measurements were made on the midsagittal sections where the disc had its maximum length, anteriorly from the junction of anterior band attachment and posteriorly from the junction of posterior band attachment (Fig. 1b).

-Joint Space:

The joint space was measured in sagittal slices from the superior most aspect of the condylar head to the most concave portion of the glenoid fossa (Fig. 1c, Fig. 2).

**Figure 3:**
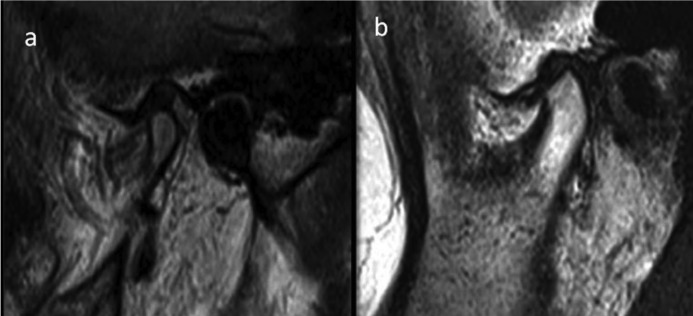
a): MRI of control showing normal appearance of condylar head. (b): MRI of OSMF patient showing flattening of condylar head.

-Condyle:

Condyle was assessed for bony changes which included flattening, erosion and advanced degenerative changes (Fig. 3 a,b).

-Statistical analysis:

The collected data was subjected to statistical analysis using IBM.SPSS version 23. To describe about the descriptive statistics, mean and S.D were used. Significant difference between the bivariate samples in independent groups was analyzed using Unpaired sample t-test. The one way ANOVA with Tukey’s Post-Hoc test was used for multi variate analysis. To find the significance in categorical data Chi-Square test was used. Pearson’s correlation was used to assess the relationship between the variables. The results were considered statistically significant at *p*<0.05 and highly significant at *p*<0.01.

## Results

The present study consisted of 40 TMJs of 20 healthy controls and 40 TMJs of 20 OSMF patients. The control group (Group C) consisted of 18 males and 2 females with a mean age of 34.8±8.79 years. The OSMF Group (Group P) consisted of 18 males and 2 females with a mean age of 37.7±10.51 years. On comparing mean age of Group C and Group P using Unpaired sample t-test, the p-value was 1.77 which was not significant, hence the controls being age and gender matched. The mean mouth opening was significantly reduced in Group P (20.73±6.00mm) when compared to Group C (43.62±5.95mm), *p*<0.01. In Group P, 14 joints were under Group II, 16 joints were under Group III and 10 joints were under Group IV.

-Thickness of articular disc:

The mean thickness of the articular disc in all the three zones i.e anterior band, intermediate zone and posterior band was significantly reduced in OSMF patients (2.27±1.04mm, 1.42±0.66mm, 2.20±1.05mm) when compared to the control group (3.53±0.86mm, 2.08±0.87mm, 3.29±1.01mm), *p*<0.01 ( Table 1). On comparison of the disc thickness between controls and the different groups of OSMF it was noted there was a significant reduction in disc thickness in Group III and Group IV OSMF patients when compared to the controls (*p*<0.01). However no significant difference was observed on comparing Group II with controls (*p*>0.05) ( Table 2, Table 3). The reduction in mouth opening was correlated with disc thickness. As the mouth opening decreases, the thickness of the articular disc decreases in all three zone (Anterior Band, Intermediate Zone, Posterior Band). The Pearson’s correlation r=+ 0.640 (anterior band), +0.449(intermediate zone), +0.622(posterior band) indicates a strong positive correlation for anterior and posterior band and moderate positive correlation for intermediate zone.

**Table 1:**
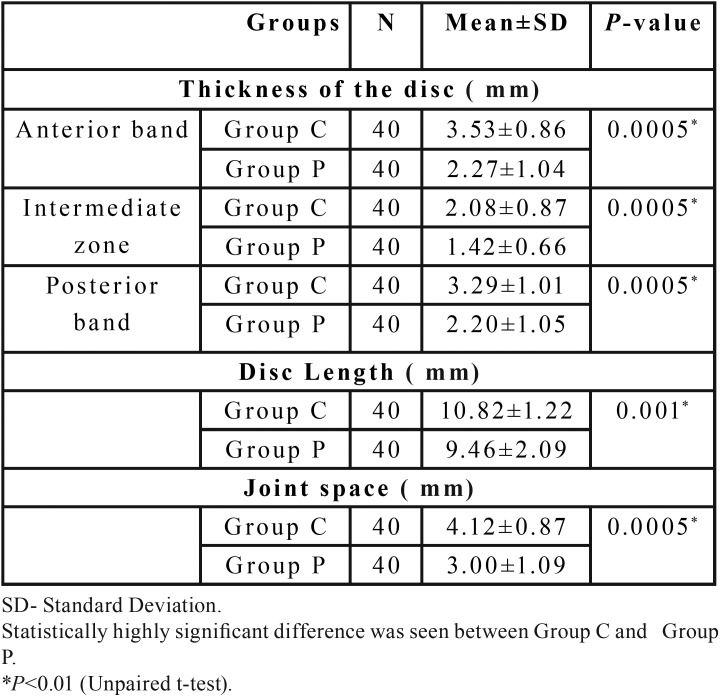
Components of temporomandibular joint assessed in Group C and Group P.

**Table 2:**
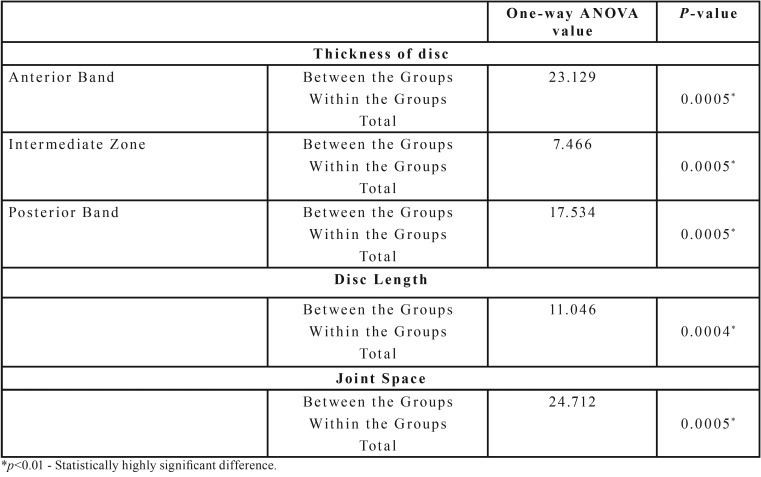
Comparison of mean disc thickness, disc length and joint space of Group II, Group III and Group IV with that of control group (One-way ANOVA).

**Table 3:**
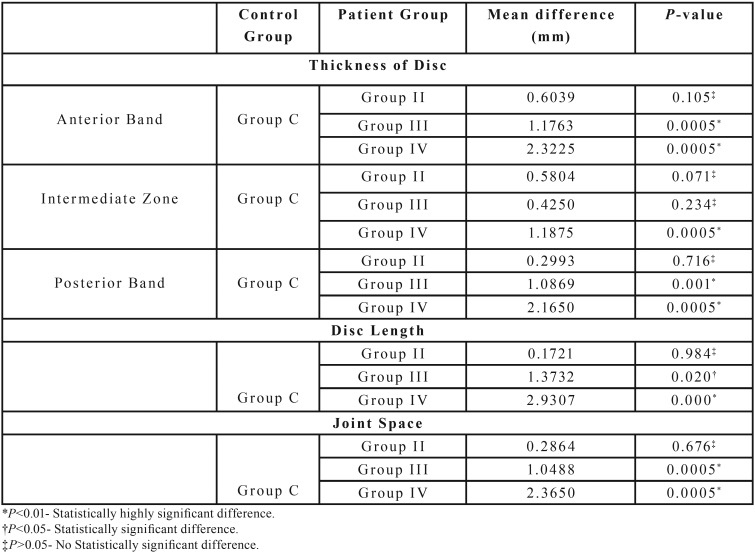
Comparison of mean disc thickness, disc length and joint space of Group II, Group III and Group IV with that of control group (Multiple comparisions- Tuckey’s Post Hoc test).

-Disc length:

There was a highly significant reduction in disc length in OSMF patients (9.46±2.09mm) when compared to controls (10.82±1.22mm), *p*<0.01 ( Table 1). On comparison of length of the disc between controls and different groups of OSMF, it was noted there was a significant reduction in disc length in Group III and Group IV OSMF patients when compared to the controls (*p*<0.05), whereas no significant difference was observed on comparing Group II with controls (*p*>0.05) ( Table 2, Table 3). There was a moderate positive correlation between reduction in mouth opening and reduction in disc length (r=0.444) which was highly significant.

-Joint space:

Joint space in OSMF patients (3.0±1.09mm) showed highly significant reduction when compared to controls (4.12±0.87mm), *p*<0.01 ( Table 1). When the mean values of the joint space of control group was compared with different groups of OSMF patients, it was noted that there was highly significant difference between controls and Group III and Group IV patients (*p*<0.01) whereas no significant difference was observed when controls were compared with Group II (*p*>0.05) ( Table 2, Table 3). As the mouth opening reduces, there is subsequent narrowing of joint space with a strong positive correlation (r=+0.622).

-Condylar changes:

The condylar changes were compared in Group C and Group P. All the 40 joints in Group C had normal condylar morphology whereas in Group P 25 joints (62.5%) had normal morphology of the condyle, 14 joints (35%) had condylar flattening and one joint(2.5%) had flattening with erosion ( Table 1). On comparison between the controls and various groups of OSMF it was noted that condylar flattening was observed only in Group III (56.3%) and Group IV(50%) OSMF patients. In addition one joint in Group IV (2.5%) also had condylar flattening with erosion. No condylar flattening was noted in Group II OSMF patients and controls. Chi-Square test to determine the significance between the Groups and within the groups was highly significant, *p*<0.01 ( Table 4).

**Table 4:**
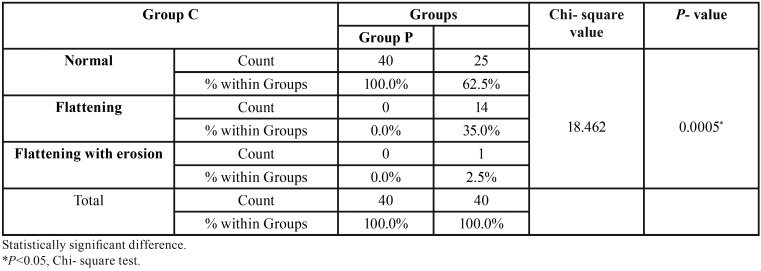
Condylar changes observed in Group C and Group P.

## Discussion

The present study was carried out to determine the Temporomandibular Joint changes in Oral Submucous Fibrosis patients with varying degrees of restricted mouth opening. Very few studies conducted in humans in the past involving joint changes following immobilization have evaluated mainly the thickness of articular cartilage which has revealed significant reduction (15,16). So far in the literature only animal studies have been done to evaluate the temporomandibular joint changes following complete or partial immobilization (9-11). Of the several conditions which lead to partial to complete immobilization of the TMJ, OSMF is one entity which is frequently encountered in day to day set up. To the best of our knowledge from literature review, no studies have been performed previously to evaluate the changes in various structures of the temporomandibular joint secondary to OSMF.

The temporomandibular joint changes were assessed using Magnetic Resonance Imaging. MRI was chosen in this study because it is considered as gold standard for imaging of TMJ. It provides high resolution and enormous tissue contrast that allows complete visualization of joint anatomy especially the soft tissues with a sensitivity and specificity of 0.86 and 0.63 respectively (5,17,18). All the images in this study were obtained in closed mouth with mandible in relaxed position because the shape and the thickness of the disc were best evaluated only in closed mouth position (19).

The mean thickness of the articular disc in all the three zones i.e anterior band, intermediate zone and posterior band was significantly reduced in OSMF patients when compared to the control group. Though no earlier studies have assessed the articular disc thickness of TMJ, the results of this study were in par with the study conducted by Vanwanseele *et al.* in 2006 where there was significant reduction in the thickness of patellar cartilage and medial tibia cartilage in the knee joints of spinal cord injured patients who had developed subsequent limitation in movement at an interval of 6, 12 and 24 months post injury(15). On comparison of the disc thickness between controls and the different groups of OSMF it was noted there was a significant reduction in disc thickness in Group III and Group IV OSMF patients when compared to the controls and the Pearson’s correlation was strong for anterior band, posterior band and moderate for intermediate zone. This signifies that changes in the disc are seen in later stages of the disease with increasing severity of reduction in mouth opening.

A significant reduction in disc length in Group III and Group IV OSMF patients compared with the controls could be due to the reduced gliding movement of the disc between condylar head and articular eminence as a consequence of trismus (20). There was a moderate positive correlation between reduction in mouth opening and reduction in disc length. These findings inferred that the length of the disc was reduced in later stages of the disease with progressive limitations in mouth opening.

A highly significant difference in the measurement of joint space was observed between controls and Group III and Group IV patients, whereas no significant difference was observed when controls were compared with Group II. As the mouth opening reduces, there is subsequent narrowing of joint space with a strong positive correlation. This implies that the Joint space gets narrowed in later stages of the disease with advanced trismus. The results were in par with the study conducted by Akeson in 1965 where there is development of joint contractures following four to twelve weeks of immobilization (21). The narrowing of joint space could be due to reduction in glycosaminoglycans, hyaluronic acid and reduced water content in the joint space following a deficient physical stimulus of motion to the joint (2-4).

Condylar flattening was observed only in Group III(56.3%) and Group IV(50%). In addition one joint in Group IV (2.5%) also had condylar flattening with erosion. No condylar flattening was noted in Group II and controls. This infers that bony changes occur only in the advanced stages of the disease due to progressive limitation in mouth opening. This could be due to the fact that as a consequence of restricted joint movement, there is generalized osteoporosis of cancellous bone and the invasion of subchondral bone by primitive mesenchymal tissue which proliferates from marrow spaces (2,3).

To the best of our knowledge, no studies have yet evaluated the temporomandibular joint changes as a consequence of trismus in OSMF patients. In spite of a paucity of proper data available with regard to reference values for various structures like disc thickness and disc length, joint space etc and a relatively limited sample size, the results have clearly addressed the objectives of the study. Future studies with larger sample size can further strengthen the results of the present study.

The following conclusions were arrived from the results of the study.

• A statistically highly significant changes in the components of temporomandibular joint namely thinning of articular disc, reduction in disc length, narrowing of joint space and condylar changes in OSMF patients with varying degrees of restriction in mouth opening when compared to controls.

• Also the severity of the changes increases with increase in severity of the disease, which was found to be statistically highly significant.
